# A relation to predict the failure of materials and potential application to volcanic eruptions and landslides

**DOI:** 10.1038/srep27877

**Published:** 2016-06-16

**Authors:** Shengwang Hao, Chao Liu, Chunsheng Lu, Derek Elsworth

**Affiliations:** 1 School of Civil Engineering and Mechanics, Yanshan University, Qinhuangdao, China; 2 The State Key Laboratory of Nonlinear Mechanics, Institute of Mechanics, Chinese Academy of Science, Beijing, China; 3 Department of Mechanical Engineering, Curtin University, Perth, WA 6845, Australia; 4 Energy and Mineral Engineering, G^3^ Center, and EMS Energy Institute, Pennsylvania State University, University Park, Pennsylvania, USA

## Abstract

A theoretical explanation of a time-to-failure relation is presented, with this relationship 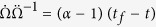 then used to describe the failure of materials. This provides the potential to predict timing (*t*_*f*_ − *t*) immediately before failure by extrapolating the trajectory 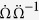 as it asymptotes to zero with no need to fit unknown exponents as previously proposed in critical power law behaviors. This generalized relation is verified by comparison with approaches to criticality for volcanic eruptions and creep failure. A new relation based on changes with stress is proposed as an alternative expression of Voight’s relation, which is widely used to describe the accelerating precursory signals before material failure and broadly applied to volcanic eruptions, landslides and other phenomena. The new generalized relation reduces to Voight’s relation if stress is limited to increase at a constant rate with time. This implies that the time-derivatives in Voight’s analysis may be a subset of a more general expression connecting stress derivatives, and thus provides a potential method for forecasting these events.

Predicting the time-to-failure of brittle materials is a long-standing problem. To describe the behavior of a material in its terminal stage of failure, Voight^1^,^2^ proposed a simple relation between the first and second derivatives of an observable quantity Ω, that is


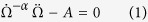


where *A* and *α* are empirical constants^2^,^3^,^4^, and the superscripted dot refers to differentiation with respect to time.

This equation (1) has been applied to changes in precursory rates of seismic energy release, ground deformation and seismic event rate^1^,^2^,^3^,^4^,^5^,^6^,^7^,^8^. It has been shown that volcanic dome-building episodes commonly exhibit acceleration in both effusive discharge rate and seismicity before explosive eruptions^9^,^10^. These kinds of accelerating behaviors of strain and seismicity appear ahead of many failure phenomena from volcanoes^2^,^5^,^6^,^7^,^8^,^11^,^12^,^13^ and landslides^14^,^15^,^16^,^17^, to laboratory samples^10^,^18^,^19^ and suggest that these precursory signals could be the basis for the application of material-failure-forecasting methods (FFM)^2^,^3^,^5^,^6^,^8^,^10^,^11^,^12^,^20^,^21^,^22^.

In the general case of *α* ≠ 1, solutions to equation (1) involving positive acceleration take the form of a power-law increase in the rate of precursory signals with time^2^,^7^,^21^. The exponent *α* is usually ~2, and *α* = 2 represents a linear relation between the reciprocal rate and time. In practice, different mechanisms or loading conditions may cause recognizable changes in the reciprocal rate curve^1^ and observed values of *α* can also fall outside these narrow limits^21^. When the exponent *α* ranges between 1 and 2^3^,^4^, the curve of the inverse velocity versus time is concave, otherwise it is convex (*α* > 2).

In the case of α = 2 , the time of failure can be estimated by extrapolating the curve of the inverse rate versus time to the time at which the inverse rate is equal to zero^2^,^7^,^20^. For other values of *α* > 1, approximate predictions may be made by other graphical extrapolation methods^20^. The accuracy and precision of forecasts using this method based on model-fitting techniques have been widely discussed^16^,^23^,^24^,^25^.

Kilburn^4^,^26^ explained the emergence of Voight’s relation by applying statistical mechanics to rock fracture. Many models based on laboratory and field data have focused on precursory behavior during deformation under constant stress. Kilburn^26^ proposed a model to extend analyses to deformation under an increasing stress. This indicated that precursory signals are controlled by an increase in applied stress, rather than by creep deformation under constant stress - thus providing an alternative to Voight’s expression, now accommodating changes with stress.

It is worth noting that it is usually required to fit the value of *α* to predict time of failure by using this empirical equation (1). However, the fluctuation of *α* usually has a significant influence on the accuracy and precision of forecasts. Also, the empirical equation (1) is usually restricted to describe stress-rate-dependent material failure, although it might also be suitable (at least approximately) to predominantly stress-rate-independent failure such as fatigue^1^. In this paper, we derive a relation to describe materials failure, and verify it by using data sets accommodating volcanic eruption and creep failure. The well-known fiber bundle model is applied to model a heterogeneous brittle stress-rate-independent material. This material is subject to a monotonically increasing stress, with the relation explored to gain insight into the conditions for material failure that are not immediately evident from using time variations alone. Based on this model, an asymptotic analysis and also Monte Carlo simulations are performed to confirm the relation close to failure.

## Theoretical derivations

Equation (1) can be simplified by noting that 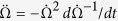, so that, substituting for 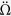 yields (supplementary information I)


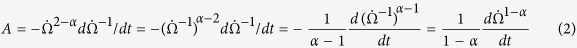


for *α* > 1, and


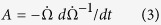


for *α* = 1. The units of *A* show a dependence on the value of α. Noting that *A* = 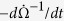 for *α* = 2 indicates that the parameter *A* has the units of 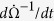 and thus reflects a characteristic scale for Ω, which, in turn, is linked to the volume of rock deformed and so will change on a case-by-case basis. This can be illustrated by the three application data sets presented by Voight^2^, which exhibit a uniform value of *α* = 2 but different *A*. Consequently, the parameter *A* represents the specific behavior of an event together with the prediction of failure time.

Since 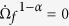 when α > 1, Equation (2) can be integrated to yield


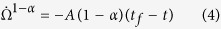


which, substituting for *A* into Equation (1) yields


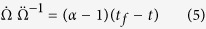


for *α*  > 1, where *t*_*f*_ is the failure time. When α = 1, Equation (1) leads directly to 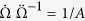.

**Figure 1:**
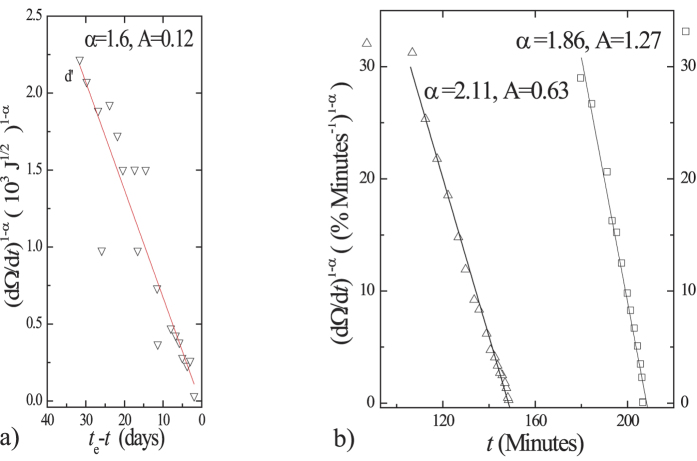
Relation between 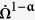 and time nearby failure. (**a**) Seismic energy release^2^ before eruption time (*t*_e_), Bezymyanny Vocano, 1960. Ω = cumulative strain release in units of 10^3^ J^1/2^. (**b**) Creep strain of soils (Hanley clay) in compression^1^,^28^.

**Figure 2:**
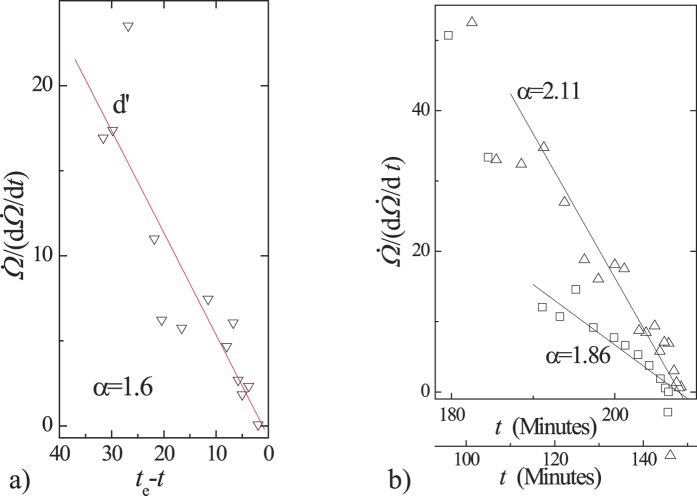
Relation between 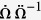 and time near failure. (**a**) Seismic energy release, Bezymyanny Volcano, shown in Fig. 1a. (**b**) Creep strains of Hanley clay shown in Fig. 1b.

Equation (4) is verified by the nearly linear behavior of 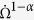 with time and close to failure for different observations from one eruption^2^ at Bezymyanny Volcano, USSR, 1960, and for creep failures^2^,^28^ shown in Fig. 1. Equation (5) indicates that 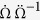 linearly decreases with time with a slope *α* − 1. The time of failure can be predicted by extrapolating the line of 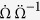 against time to a point at which 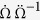 is equal to zero, as for the three events shown in Fig. 2. It is of particular interest that this gives a method to predict failure time before failure without fitting the values of *α* and *A*, for α > 1.

### Model analysis

In this section, a model is developed to show an alternative expression for Voight’s relation (1) based on changes with stress for a “stress-rate independent” material defined as the case where stress in the material is independent of the strain rate.

### Model

We consider a statistical fiber-bundle model with *N* fibers connected in parallel, clamped by a rigid yoke at both ends, and extended by an applied longitudinal stress. Here, a global load-sharing criterion is enforced by the rigid yoke, requiring redistribution of load between remaining fibers following the failure of one or more of the fibers. In this form some closed form analytic results can be obtained.

For a continuous case (or in the limit of infinite *N*), the constitutive relationship can be expressed as^27^ (Supplementary Information, II)


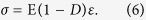


Here *σ* = applied stress, E = Young’s Modulus, *ε = *strain. The damage fraction, *D*, ranges from zero to unity. For quasistatic failure or the case where stress is independent of the strain-rate, the damage variable *D* can be expressed uniquely by the distribution of strain (or stress) thresholds of elements. This can be described by a uniform distribution *D*(*ε*) = *ε* between 0 and 1 for the purposes of explicit calculations, or a Weibull distribution of the form 
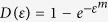
. This latter form is widely used to describe how local breaking strengths are distributed within a material^29^ and is convenient in the analysis, due to its versatility (Supplementary Information, III).

### Analytic derivation

**Figure 3:**
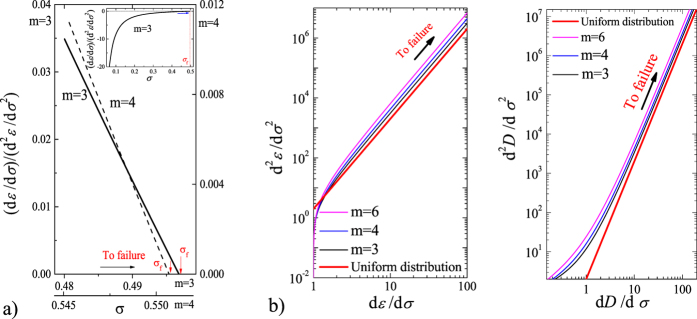
Analytical results of strain and damage evolution. (**a**) Relation between ((*dε*)/(*dσ*))((*d*^2^*ε*)/(*dσ*^2^))^−1^ and stress nearby failure for a Weibull distribution with *m* = 3 and 4. The approximately linear relation with a slope 2 = *β* − 1 verifies relation (5). Insert for *m* = 3 is to show the tendency of ((*dε*)/(*dσ*))((*d*^2^*ε*)/(*dσ*^2^))^−1^ evolving to failure. (**b**) The relation between *dε*/*dσ* (or *dD*/*dσ*) and *d*^2^*ε*/*dσ*^2^ (or *d*^2^*D*/*dσ*^2^). *dε*/*dσ* (or *dD*/*dσ*) and *d*^2^*ε*/*dσ*^2^ (or *d*^2^*D*/*dσ*^2^) are the first and second derivatives of strain (or damage) with respect to stress, respectively. The straight red line represents a uniform strength distribution and has a slope of 3, with the remaining relations corresponding to a Weibull distribution with *m* = 3, 4, and 6, respectively.

For a stress-rate-independent material, the relative change of measurable responses such as damage *D* and strain *ε*, with respect to the controlling variable *σ*, are most useful in its application. The first and second derivatives of damage *D* and strain *ε* are calculated with respect to the controlling variable *σ*: *dε*/*dσ* (or *dD*/*dσ*) and *d*^2^*ε*/*dσ*^2^ (or *d*^2^*D*/*dσ*^2^), and are shown in Fig. 3. The fiber system will fail completely when the load reaches the maximum stress, so the critical failure stress 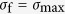 and can be analytically derived by using the condition of *dσ*/*dε*|_*f*_ = 0. For example, this gives *σ*_f_ = 1/4 for the uniform distribution and *σ*_f_ = (*me*)^−1/*m*^ for the Weibull distribution.

The relation between (*dε*)/(*dσ*)((*d*^2^*ε*)/(*dσ*^2^))^−1^ and stress becomes linear as failure is approached (see Fig. 3a) and shows a relation (*dε*)/(*dσ*)((*d*^2^*ε*)/(*dσ*^2^))^−1^ = 2(*σ*_f_ − *σ*), which is similar to equation (5). A slope of 2 indicates that the critical exponent is 3, which is consistent with the results shown in Fig. 3b. Based on an observed linear dependence (the right portion of the curves in Fig. 3b), the increase in response ahead of failure can be described as *d*^2^*ε*/*dσ*^2^ = *k*(*dε*/*dσ*)^*β*^ or *d*^2^*D*/*dσ*^2^ = *k*(*dD*/*dσ*)^*β*^ with *β* = 3. For both the Weibull distribution with different shape parameters *m* and for the uniform distribution, all cases present the same exponent of 3 but exhibit different values of *k* (see Fig. 3b). Furthermore, for the Weibull distribution, a larger Weibull index *m* represents a larger value of *k*. These imply that the power exponent exhibits a common characteristic during the failure process. However, the parameter *k* reflects their sample-specific behavior.

We now present an analytic derivation of these relations. The stress-strain equation (6) implies that the stress *σ* can be expressed as a function of strain: *σ*(*ε*)* = E*[1 − *D*(*ε*)]*ε*, and that therefore


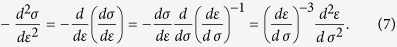


The analogous procedure for expressing stress as a function of damage: *σ* = *σ*(*D*) leads to


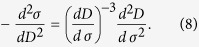


This confirms that the critical parameters are *β* = 3 and *k* = −(*d*^2^*σ*/*dε*^2^)_*f*_ near failure.

To clearly demonstrate the characteristics of equations (7) or (8) and the tendencies of parameters *k* and *β*, we turn to the expansion of *σ*(*ε*) as a function of *ε* in the vicinity of the failure point *σ*_f_. That is


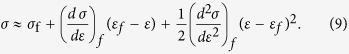


Substituting (*dσ*/*dε*)_*f*_  = 0 into equation (9), we get


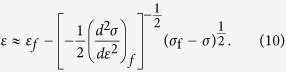


When close to failure, then


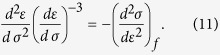


An analogous procedure may be applied to calibrate in terms of damage *D* by noting that strain can be expressed as a function of *D*, for example *ε* = *D* for a uniform distribution and *ε* = [−log(1−*D*)]^1/*m*^ for a Weibull distribution. This also leads to


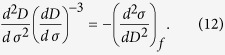


Thus, equations (11) and (12) give a similar expression to Voight’s relation (1), i.e.


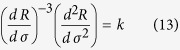


where *R* represents the corresponding response variable such as strain and damage in this paper when the system is loaded by controlling stress, *k* = −((*d*^2^*σ*)/(*dR*^2^))_*f*_. Here, the critical exponent of 3 is independent of the specific expression *σ* *=* *σ* (*ε*), but *k* is determined by the specific stress-strain relationship of a sample. For the uniform strength distribution, *k* = 2 and for the Weibull strength distribution, the parameter *k* = *m*^1+1/*m*^*e*^−1/*m*^ is a function of the Weibull index *m*. It should be mentioned that, if (*d*^2^*σ*/*dR*^2^)_*f*_ tends to infinity or zero, equations (9)–(13) give *β* less than 3. So, *β* = 3 should be the upper limit of *β*. It is not evident that equations (13) and (1) should be equivalent, because they were determined for different sets of loading conditions^26^. In this paper, we focus on a load condition with increasing stress. Thus, we use the symbol *β* other than *α* in Voight’s original relation (1) to represent the critical exponent.

By performing the first and second differentiation on expression (10) and rearranging, we obtain a similar expression to equation (5) as


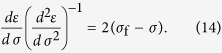


Similarly, we obtain


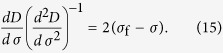


This confirms the linear relation of (*dR*)/(*dσ*)((*d*^2^*R*)/(*dσ*^2^))^−1^ with *σ* nearby failure, as shown in Fig. 3b.

### Numerical analysis

It is important to note that the preceding analytical derivations are exact only in the limit for a continuous case where the total number of fibers, *N,* is infinite. To further examine the response of equations (13) and the critical parameters for the non-continuous (discrete) case, Monte Carlo simulations of the failure process were performed. For simplicity, the Young’s modulus *E* is set to unity, with all stresses normalized by *E*. As the load on a bundle of *N* fibers monotonically increases, simulations of the failure process proceed as follows:

1. Breaking thresholds are randomly chosen according to a probability distribution with the thresholds arranged in increasing order.
2. Load is applied and incremented with each step by the minimum required to break the next fiber. Each broken fiber sheds its load that is then redistributed equally to all surviving fibers.
3. The revised load on all surviving fibers as well as the nominal strain is recalculated.
4. This process is repeated until the actual load on all surviving fibers is less than their individual thresholds.
5. Return to step (1) and repeat the process until the entire bundle fails.

**Figure 4:**
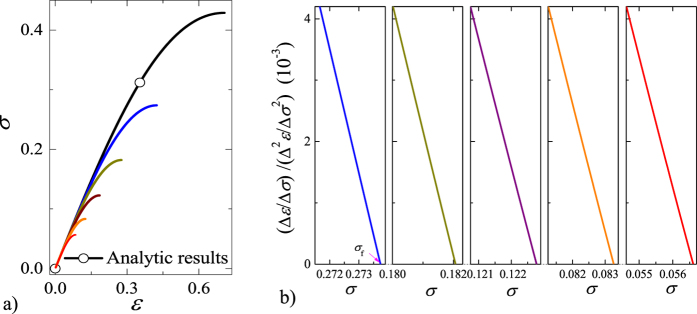
Simulation results of *σ*–*ε* curves and critical behaviors of ((Δ*ε*)/(Δ*σ*))((Δ^2^*ε*)/(Δ*σ*^2^))^−1^. Weibull distributions with *m* = 2 and *N* = 10^4^ fibers are used in each sample. (**a**) Numerical curves of the nominal stress versus strain. (**b**) Almost linear relations between ((Δ*ε*)/(Δ*σ*))((Δ^2^*ε*)/(Δ*σ*^2^))^−1^ and stress nearby failure shown for all samples.

In all simulations, (Δ*R*)/(Δ*σ*)((Δ^2^*R*)/(Δ*σ*^2^))^−1^ presents a common linear relationship with stress as failure is approached (see Fig. 4b), although individual failure stresses are different (see Fig. 4a). In the case of discrete simulations, the discrete derivative operator “∆” is substituted for the continuous derivative operator “d”.

**Figure 5:**
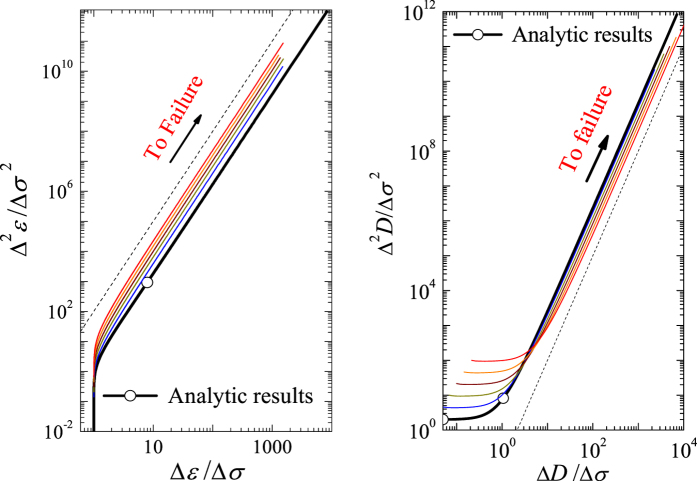
Simulation results of Δ^2^*R*/Δ*σ*^2^ versus Δ*R*/Δ*σ* for 5 samples shown in Fig. 4. Δ*ε*/Δ*σ* (or Δ*D*/Δ*σ*) and Δ^2^*ε*/Δ*σ*^2^ (or Δ^2^*D*/Δ*σ*^2^) are the first and second derivatives of strain (or damage) with respect to stress, respectively. The dashed line of slope 3 is included as a visual guide.

A linear relationship in the terminal stage of failure, shown in Fig. 5, validates the proportionality between the logarithm of 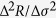 and the logarithm of Δ*R*/Δ*σ*. The continuous case has a highest maximum stress (see Fig. 4a) and a smaller value *k* in comparison to the discrete cases, but all samples exhibit the same exponent of *β* = 3 (see Fig. 5). It is worth noting that samples in the discrete case have a different value of *k* even for an identical Weibull index (*m* = 2). A lower peak stress implies a lower value of *k*, suggesting that *k* reflects sample-specific behavior, whereas the exponent *β* is universal.

For the continuous case, the load condition is analogous to a load increasing at a constant rate. In the numerical analysis, the load increment applied at each step is the minimum required to break the next fiber, and the load rate does not need to be constant. Thus these relationships are actually independent on any particular style of loading rate and are functionally loading-rate-independent. This indicates that equation (13) can be applied to a broader suite of monotonic loading conditions than merely a linearly increasing load.

## Discussion and Conclusions

Based on Voight’s relation 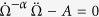, a new relation 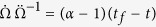 is proposed by a general derivation for α > 1. The revised relation yields a new method for forecasting the time of failure by linearly extrapolating 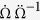 with time to zero, without the need to fit the exponent *α*. In application of this method, it is both feasible and preferable that the two methods are used together to check that the trend inferred from one method is confirmed by the other.

Equation (13) describes the failure of a material, in which the stress is fully independent of the strain rate. This is equivalent to Voight’s relation (1) if the stress is increased at a constant rate with time, such that d*R/*d*σ* ∝d*R/*d*t*. Thus, this result suggests that the time-derivatives in Voight’s analysis might be a subset of a more general expression connecting stress derivatives. In the present paper, this general relation can be expressed as ((*dR*)/(*dσ*))^−*β*^((*d*^2^*R*)/(*dσ*^2^)) = *k* (*R* represents dependent variables such as damage and strain in this paper or could be analogous parameters as seismic activity (e.g. RSAM))^5^. This implies that Voight’s original relation (1) does not hold for any chosen parameter^26^, for example equation (13) can only work for those which can be directly related to d*R*/d*σ*. In some cases for eruptions^26^, the precursory trends were controlled by an increase in applied stress, rather than creep failure. So, the present results provide an alternative relation ((*dR*)/(*dσ*))^−*β*^((*d*^2^*R*)/(*dσ*^2^)) = *k* to describe the failure by using changes with stress and thus a potential way for forecasting these events.

In equation (13), the parameter *k* changes for different samples (e.g., different size, or constitutive parameters) even if it has a same expression of *k*. For example, the calculated cases for a Weibull distribution of strengths exhibit different *k* values even though these calculation samples have the same Weibull modulus *m* and *k* has the same expression *k* = −*d*^2^*σ*/*dR*^2^.

## Additional Information

**How to cite this article**: Hao, S. *et al.* A relation to predict the failure of materials and potential application to volcanic eruptions and landslides. *Sci. Rep.* **6**, 27877; doi: 10.1038/srep27877 (2016).

## Supplementary Material

Supplementary Information
