# Supplementary material for: A relation to predict the failure of materials and potential application to volcanic eruptions and landslides
Source: Sci Rep. 2016 Jun 16;6:27877. doi: 10.1038/srep27877 (PMC4910060; doi:10.1038/srep27877)
Supplement: Supplementary Information [file srep27877-s1.pdf]

# Supplementary Information

## A relation to predict the failure of materials and potential application to volcanic eruptions and landslides


Shengwang Hao[1,2], Chao Liu[1], Chunsheng Lu[3], and Derek Elsworth[4,*]

[1]School of Civil Engineering and Mechanics, Yanshan University, Qinhuangdao, China

[2]The State Key Laboratory of Nonlinear Mechanics, Institute of Mechanics, Chinese Academy of Science, Beijing, China

[3]Department of Mechanical Engineering, Curtin University, Perth, WA 6845, Australia

[4]Energy and Mineral Engineering, G[3] Center, and EMS Energy Institute, Pennsylvania State University, University Park, Pennsylvania, USA.

*Correspondence to: elsworth@psu.edu


## I. An alternative derivation of equation (2) to demonstrate the characteristics of parameter A

The response variable $\Omega$ can be written as a function of time $t$: $\Omega = \Omega(t)$, then $\ddot{\Omega}$ can be expressed as

$$\ddot{\Omega} = \frac{d^2\Omega}{dt^2} = \frac{d}{dt}\left(\frac{d\Omega}{dt}\right) = \frac{d}{dt}\left(\frac{dt}{d\Omega}\right)^{-1} = -\left(\frac{dt}{d\Omega}\right)^{-2}\frac{d}{dt}\left(\frac{dt}{d\Omega}\right), \tag{A1}$$

and therefore

$$\dot{\Omega}^{-2}\ddot{\Omega} = -\frac{d}{dt}\left(\frac{dt}{d\Omega}\right) = A. \tag{A2}$$

Thus, $\alpha$ = 2 presents an expression of $A = -d\dot{\Omega}^{-1}/dt$, which can also be derived by using equations (3b) in Voight[2] and (4) in Voight[1]. The expression for $A$ shows that $A$ is merely the negative slope of the curve of the inverse velocity versus time and tends to a critical constant since $\alpha$ = 2 suggests that the reciprocal rate curve with time is linear nearby failure.

Rearranging equation (A3), $A$ can be generally expressed as $A = -\dot{\Omega}^{2-\alpha} d\dot{\Omega}^{-1}/dt$, and further derived as

$$A = -\dot{\Omega}^{2-\alpha} d\dot{\Omega}^{-1}/dt = -\left(\dot{\Omega}^{-1}\right)^{\alpha-2} d\dot{\Omega}^{-1}/dt = -\frac{1}{\alpha-1}\frac{d\left(\dot{\Omega}^{-1}\right)^{\alpha-1}}{dt} = \frac{1}{1-\alpha}\frac{d\dot{\Omega}^{1-\alpha}}{dt} \tag{A3}$$

for $\alpha > 1$. $A = -\dot{\Omega}\, d\dot{\Omega}^{-1}/dt$ for $\alpha$ = 1. So, the Voight[1,2] relation $\dot{\Omega}^{-\alpha}\ddot{\Omega} - A = 0$ or relation (A3) is valid throughout the entire lifetime of materials – not merely at the point of incipient failure. In this, the parameter $A$ is a variable but tends to a critical constant nearby failure.

## II. Constitutive equation for fiber bundle model

A statistical fiber bundle model with $N$ fibers connected in parallel (clamped at both ends) is considered and a load $\sigma$ is applied at one end (as illustrated in Figure 1). All fibers have a same Young's modulus $E$. Here, a global load-sharing criterion is chosen for the redistribution of load following the failure of one or more of the fibers as in this form some closed analytic results can be obtained.

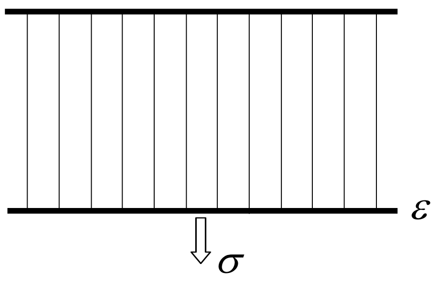


Figure A1. A statistical fiber bundle model with $N$ parallel fibers, where the load is applied to the rigid end platens. All the fibers are assumed to have the same Young's modulus $E$ until they break. A fiber breaks when it reaches its strength and then no longer carries any load. The surviving fibers equally share the force released by the broken fibers.

Before it breaks, a fiber extends as a linearly elastic member

$$\sigma_t = E\varepsilon\,, \tag{A4}$$

where $\sigma_t$ is the true stress on the fiber and $\varepsilon$ is the strain. When the stress reaches its strength threshold, the fiber breaks and sheds the load. The surving fibers equally share the stress released by the broken fiber. If the system is subjected to a stress $\sigma$ (figure A1), the true stress on a surviving fiber is

$$\sigma_t=\sigma/(1-D), \tag{A5}$$

where $D$ represents the damage fraction that is defined as $D=N_b/N$, with $N_b$ the number of broken fibers. For a continuous case (or in the limit of infinite $N$), when the fibers have a true stress $\sigma_t$, the damage fraction can be expressed as

$$D\left(\sigma_t\right) = \int_0^{\sigma_t} h(\sigma_c)\,d\sigma_c\,. \tag{A6}$$

Where $\sigma_c$ is the stress threshold of the individual fiber elements and $h(\sigma_c)$ is the probability density function of thresholds. From equation (A4), equation (A6) can also be expressed as

$$D(\varepsilon) = \int_0^{\varepsilon} h(\varepsilon_c)\, d\varepsilon_c \,. \tag{A7}$$

where $\varepsilon_c$ is the strain threshold of the fiber elements and $h(\varepsilon_c)$ is the probability density function of those strain thresholds. According to equations (A4), (A6) and (A7), the constitutive relationship can be obtained as

$$\sigma = E[1-D(\varepsilon)]\varepsilon. \tag{A8}$$

If the Young's modulus $E$ is set to a unit value, which means that the stresses are normalized with $E$, then

$$\sigma = [1-D(\varepsilon)]\varepsilon. \tag{A9}$$

## III. Derivation of the critical stress for a Uniform distribution and Weibull distribution of strengths

The fiber system will totally fail when the load reaches its maximum stress, i.e. its limit load capacity. Thus, the maximum stress point $\sigma_{\max}$ is the critical point of failure $\sigma_{\mathrm{f}}$ and it satisfies that

$$d\sigma/d\varepsilon\big|_f = 0 \,. \tag{A10}$$

From equation (A7), the derivative of stress with respect to strain is

$$\frac{d\sigma}{d\varepsilon} = 1 - D(\varepsilon) - \varepsilon \frac{dD}{d\varepsilon} \,. \tag{A11}$$

For a uniform distribution, $D(\varepsilon) = \varepsilon$, then we get

$$\frac{d\sigma}{d\varepsilon} = 1 - \varepsilon - \varepsilon \,. \tag{A12}$$

Substituting into equation (A10), we then get $\varepsilon_{\mathrm{f}} = 1/2$. Substituting $\varepsilon_{\mathrm{f}} = 1/2$ into equation (A9) , the critical failure stress is $\sigma_{\mathrm{f}} = \sigma_{\max} = 1/4$.

For a Weibull distribution, $D(\varepsilon) = 1 - e^{-\varepsilon^m}$ and similarly, we get

$$\frac{d\sigma}{d\varepsilon} = (1 - m\,\varepsilon^{m})\,e^{-\varepsilon^{m}} \,. \qquad \text{(A13)}$$

Then at the maximum stress, the strain $\varepsilon_{\mathrm{f}} = (1/m)^{1/m}$ and the critical failure stress is $\sigma_{\mathrm{f}} = \sigma_{\max} = (me)^{-1/m}$.
